# Mitochondria‐Related Pathogenic Genes in Paediatric Asthma: A Multi‐Omics Mendelian Randomization Study

**DOI:** 10.1111/jcmm.71102

**Published:** 2026-03-27

**Authors:** Biyu Zhang, Yaqin Li, Bo Ding, Xiaoyan Li, Yanming Lu

**Affiliations:** ^1^ Department of Paediatrics, Renji Hospital Shanghai Jiao Tong University School of Medicine Shanghai China

**Keywords:** colocalization analysis, Mendelian randomization, mitochondrial genes, multi‐omics, paediatric asthma

## Abstract

Mitochondrial dysfunction is implicated in asthma pathogenesis, but causal roles of mitochondrial‐related genes in paediatric asthma remain unclear. We performed a multi‐omics Mendelian randomization study integrating GWAS data from paediatric asthma cohorts with blood‐based methylation quantitative trait loci (mQTLs), expression QTLs (eQTLs) and protein QTLs (pQTLs) datasets. Causal inference was assessed using Summary‐data‐based Mendelian Randomization (SMR) and HEIDI testing, complemented by colocalization analysis. Findings were validated in independent cohorts and evaluated for tissue specificity using GTEx. Functional enrichment and protein–protein interaction (PPI) network analyses were conducted. SMR analysis identified 80 methylation sites spanning 54 genes, 26 gene expressions, and three proteins significantly associated with paediatric asthma. Colocalization analysis confirmed strong evidence for 10 methylation sites (7 genes), the *STX17* eQTL (PP.H4 = 0.98) and the *UNG* pQTL (PP.H4 = 0.84). Tissue‐specific eQTL validation replicated the *STX17* association. Multi‐omics integration associated *ALAS1* (cg13241645, cg15698299) and *TXNRD1* (cg09884423) with asthma at both methylation and expression levels, with colocalization supporting both *ALAS1* associations. Furthermore, integrated mQTL‐eQTL analysis suggests that DNA methylation potentially regulates *ALAS1* and *TXNRD1* expression. Functional enrichment and network analyses revealed that these candidate genes converge on mitochondrial metabolic pathways and identified seven hub genes with potential regulatory significance (*SDHB, MFN2, GLDC, PHB2, TXNRD1, ATP5MC1* and *PHB*). This study provides multi‐omics evidence supporting a causal role for mitochondrial‐related genes, particularly *ALAS1* and *TXNRD1*, in paediatric asthma, offering new insights into pathogenesis and potential therapeutic targets.

**Abbreviations**
eQTL: expression quantitative trait locus
GO: gene ontology
GWAS: genome‐wide association study
KEGG: Kyoto Encyclopedia of Genes and Genomes
mQTL: methylation quantitative trait locus
MR: Mendelian randomization
PPI: protein–protein interaction
pQTL: proteomic quantitative trait locus
ROS: reactive oxygen species
SMR: summary data‐based Mendelian randomization

## 1 Introduction

Asthma is the most common long‐term respiratory disorder in children, posing significant challenges to their health and overall well‐being worldwide. Paediatric asthma is the most prevalent chronic respiratory disease, affecting the health and quality of life of millions of children worldwide. Its characteristic symptoms include recurrent coughing, wheezing, and shortness of breath [1], which can lead to acute asthma attacks and even be life‐threatening in severe cases. Currently, the management of paediatric asthma primarily involves the long‐term use of inhaled corticosteroids and long‐acting β2‐agonists [2], along with symptomatic treatments depending on the condition. However, these treatments are often accompanied by high treatment costs and potential side effects, limiting their widespread applicability [3, 4]. The pathophysiological processes of paediatric asthma are complex, with mitochondrial dysfunction being recognized as a central element in the pathogenesis of the disease [5]. Although prior research has identified connections between abnormal mitochondrial activity and asthma in children, the detailed molecular pathways remain insufficiently defined.

Mitochondria serve as the primary energy generators within cells, and disturbances in their function have been implicated in numerous human diseases [6]. In the context of childhood asthma, impaired mitochondrial processes play a critical role. Observed alterations include diminished activity of the electron transport chain, increased membrane permeability, uncoupling events, and disruptions in calcium homeostasis. Collectively, these mitochondrial anomalies drive excessive generation of reactive oxygen species (ROS), which can compromise cellular integrity and trigger inflammatory and remodelling responses in the airways [7, 8]. Increased mitochondrial autophagy may be a mechanism by which the body clears damaged mitochondria, but excessive autophagy could also lead to further impairment of mitochondrial function [9]. Mitochondrial dysfunction can affect the production of cytokines, for example, Thymic stromal lymphopoietin (TSLP) regulates ROS production and mitochondrial autophagy through AMPK activation and histone modification, thereby influencing the immune response in asthma patients [10]. Additionally, changes in mitochondrial DNA copy number may reflect the dynamic changes of mitochondria during asthma [11]. In terms of genetic factors, mitochondrial gene mutations and polymorphisms may increase the susceptibility to paediatric asthma [12, 13]. Therapeutic agents targeting mitochondrial pathways—such as neurokinin 1 receptor antagonists—have shown promise in preclinical models by mitigating airway inflammation and mitochondrial abnormalities through activation of the Nrf2/HO‐1 axis, resulting in improved respiratory function [14]. Despite these advances, the specific mitochondrial genes involved in the onset and progression of paediatric asthma, as well as their mechanistic roles, have yet to be fully elucidated.

Mendelian randomization (MR) is an epidemiological technique that leverages genetic variants as instrumental variables to explore potential causal relationships between exposures and diseases [15]. The integration of MR with modern genomics and bioinformatics—particularly through Summary data‐based Mendelian Randomization (SMR)—enables the systematic assessment of links between genetic factors and complex traits by combining genome‐wide association study (GWAS) findings with multi‐omics resources, such as expression (eQTL), methylation (mQTL), and proteomic (pQTL) quantitative trait loci [16]. By minimizing confounding and reverse causation, SMR provides a robust framework for causal inference. Previous MR studies of asthma have predominantly examined immune‐related or inflammatory pathways, often using single‐omics approaches that do not specifically address mitochondrial genes [17]. Likewise, existing colocalization analyses in asthma genetics have generally been genome‐wide and have not systematically targeted mitochondrial‐related genes [18].

In this study, we apply a multi‐omics MR framework to systematically identify mitochondrial‐related genes with potential causal effects on paediatric asthma, integrating GWAS, mQTL, eQTL and pQTL datasets. We further validate tissue‐specific expression profiles and employ downstream bioinformatics analyses—including functional enrichment and network modelling—to clarify the biological roles of candidate genes and uncover central regulatory elements. Collectively, these efforts aim to advance mechanistic understanding and inform more precise therapeutic strategies for paediatric asthma.

## 2 Materials and Methods

### 2.1 Study Design

**FIGURE 1:**
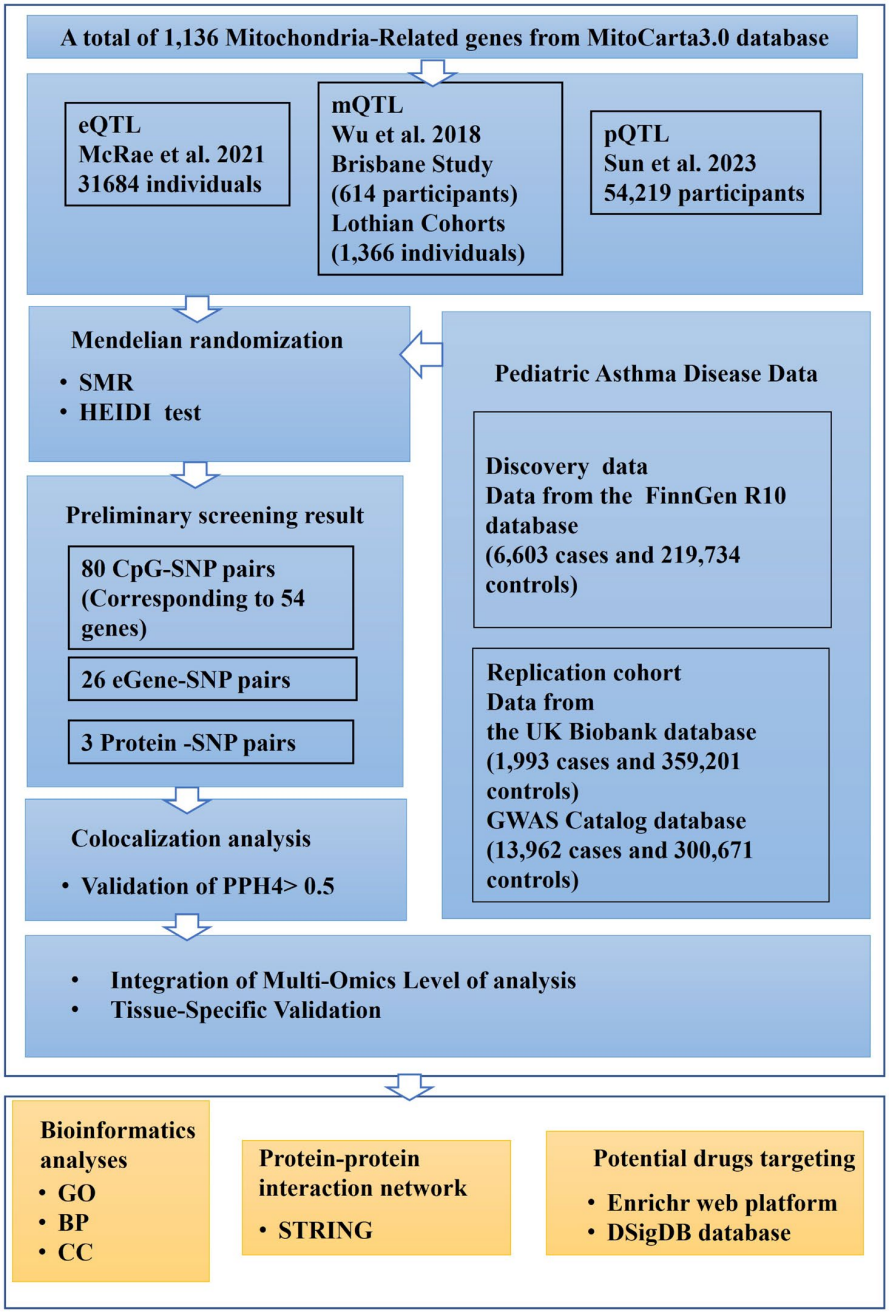
Study design flow chart.

This research followed the STROBE‐MR guidelines for reporting MR studies [19]. We applied MR approaches to systematically investigate whether mitochondrial mechanistic genes are causally linked to paediatric asthma. The workflow comprised several key steps: comprehensive genetic data extraction, application of SMR and HEIDI tests to identify associations, and subsequent colocalization analysis for pinpointing shared causal variants. Figure 1 provides an overview of the study design, variant selection process, and analytical pipeline. As all datasets were derived from publicly accessible GWAS, this study did not require institutional ethics approval.

### 2.2 Data Sources

A total of 1136 mitochondrial machinery genes were selected from the MitoCarta3.0 database. The main GWAS dataset used for discovery was the Finnish R10 cohort (Finngen_R10_ATHMA_CHILD_EXMORE), which included 6603 individuals diagnosed with paediatric asthma and 219,734 controls. Replication in independent cohort analyses was conducted using two additional resources: the GWAS Catalogue dataset (GCST007800; 13,962 cases and 300,671 controls) and the UK Biobank (UKB‐d‐ASTHMA_CHILD; 1993 cases and 359,201 controls).

Blood eQTL summary statistics were collected from the eQTLGen consortium [15], encompassing data from 31,684 participants. For blood mQTLs, we used meta‐analytic data from two European cohorts: the Brisbane Systems Genetics Study (614 participants) and the Lothian Birth Cohorts (1366 individuals) [16]. Proteomic QTLs (pQTLs) were sourced from Sun et al. [20], covering 54,219 participants from the UK Biobank.

We further incorporated tissue‐specific eQTL data from the GTEx v8 resource, which consists of 838 donors and 52 tissue types as well as two cell lines, totalling 17,382 samples. For paediatric asthma‐related analyses, lung tissue eQTLs were specifically utilized.

### 2.3 SMR and HEIDI Analysis

The SMR software package (version 1.3.1) was used to perform SMR and HEIDI tests, enabling the assessment of associations between methylation, gene expression, protein abundance, and paediatric asthma risk [16]. The MR analysis used top‐ranked cis‐QTLs as instrumental variables, focusing on those within ±1 Mb of the respective gene probe and passing a genome‐wide significance threshold (*p* < 5 × 10^−8^) [21]. Single nucleotide polymorphisms (SNPs) were excluded if allele frequency differences between datasets (including LD reference, QTL and outcome datasets) exceeded 0.2. Additionally, for mQTL, eQTL, and pQTL analyses, the proportion of SNPs with substantial allele frequency differences was limited to 0.05. Beyond examining the causal effects of QTLs (mQTL, eQTL, pQTL) on paediatric asthma, we also evaluated the influence of mQTLs on eQTLs by treating methylation as the exposure and expression as the outcome.

We also implemented a multi‐SNP SMR approach (−SMR‐multi), which considers all SNPs within a gene region that both meet the genome‐wide significance cutoff (*p* < 5 × 10^−8^) and have low linkage disequilibrium (LD *r* ^*2*^ < 0.9) with the lead SNP. Only results meeting the following criteria were retained for downstream analysis: P_SMR < 0.05, P_SMRmulti < 0.05, and P_HEIDI > 0.05. The HEIDI test (*p* > 0.05) was used to filter out signals likely due to linkage rather than pleiotropy [16]. For multiple testing correction, we applied a false discovery rate (FDR) adjustment to the SMR *p*‐values across all tested genes.

### 2.4 Colocalization Analysis

Colocalization was carried out using the R package coloc to determine whether mitochondrial gene‐related cis‐QTLs (mQTLs, eQTLs or pQTLs) and asthma GWAS loci share common causal variants [22]. Five posterior probabilities were estimated, representing the likelihood of various scenarios: H0 (no association), H1 (association with trait 1 only), H2 (trait 2 only), H3 (both traits but distinct variants), and H4 (both traits and the same causal variant). For mQTL‐GWAS, eQTL‐GWAS, and pQTL‐GWAS colocalization, a ± 1 Mb window was used. A QTL signal was considered to have strong colocalization with a GWAS locus if P12 was ≤ 5 × 10^−5^ and the posterior probability for H4 (PP.H4) ≥ 0.8; loci with moderate colocalization evidence were defined as those with 0.5 ≤ PP.H4 < 0.8 [23].

All analyses were performed in R version 4.4.1. Manhattan plots were generated using the ggplot2 and ggrepel packages, while forest plots were produced with forestplot. SMR locus and effect plots were adapted from the code published by Zhu et al. [24].

### 2.5 Functional Enrichment, Protein–Protein Interaction Network and Drug Prediction

To characterize the biological relevance of candidate genes, we applied several bioinformatic analyses to the 29 genes with significant eQTL and pQTL associations to paediatric asthma. Gene Ontology (GO) and Kyoto Encyclopedia of Genes and Genomes (KEGG) pathway enrichment analyses were performed with the R package clusterProfiler. GO analysis encompassed three aspects: biological processes (BP), cellular components (CC), and molecular functions (MF). KEGG pathways classified under ‘Human Diseases’ were excluded to focus on core biological mechanisms. All enrichment *p*‐values were adjusted using the Benjamini–Hochberg method, and terms with adjusted *p* < 0.05 were considered significant [25].

A protein–protein interaction (PPI) network was constructed using the STRING database (https://cn.string‐db.org/) and visualized in Cytoscape. The CentiScaPe plugin (v2.2) was used to identify hub genes based on degree, betweenness and closeness centrality; genes ranking in the top 10 for all three measures were defined as hub genes [26].

Finally, candidate drugs targeting these hub genes were predicted via the Enrichr platform (https://maayanlab.cloud/Enrichr/) by leveraging its integration with the DSigDB database, which provides curated gene sets for drug and small molecule perturbations [27].

## 3 Results

### 3.1 Integration of Paediatric Asthma GWAS With Blood Mitochondrial‐Related mQTL Data

**FIGURE 2:**
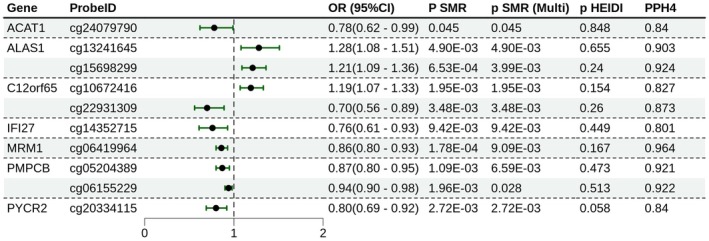
SMR analysis results for mQTLs in the Finngen_R10_ASTHMA_CHILD_EXMORE cohort (Highlighting Significant Colocalized Genes)‐ Forest plot.

SMR analysis identified 80 methylation sites (54 unique genes) significantly associated with paediatric asthma (P‐SMR < 0.05, P‐SMR multi < 0.05, P‐HEIDI > 0.05; Table S1). Among these, 28 sites (20 genes) exhibited moderate colocalization evidence (PP.H4 > 0.5; Figure S1A), while 10 sites (7 genes) showed strong evidence (PPH4 ≥ 0.8; Figure 2), including *ALAS1* (cg13241645, cg15698299), *C12orf65* (cg10672416, cg22931309), *IFI27* (cg14352715), *MRM1* (cg06419964), *PMPPCB* (cg05204389, cg06155229) and *PYCR2* (cg20334115). Specifically, cg09424348 (*VARS2*) and cg06419964 (*MRM1*) passed FDR correction (Table S1).

Exploratory replication in the GCST007800 cohort identified three associations: cg19266387 (*PARL*), cg14352715 (*IFI27*) and cg01655341 (*BCKDHA*) (Table S2). Further exploratory replication in the UKB‐D‐ASTHMA_CHILD cohort identified cg09618197 at *LYRM4* (Table S3).

### 3.2 Integration of Paediatric Asthma GWAS With Blood Mitochondrial‐Related eQTL Data

**FIGURE 3:**
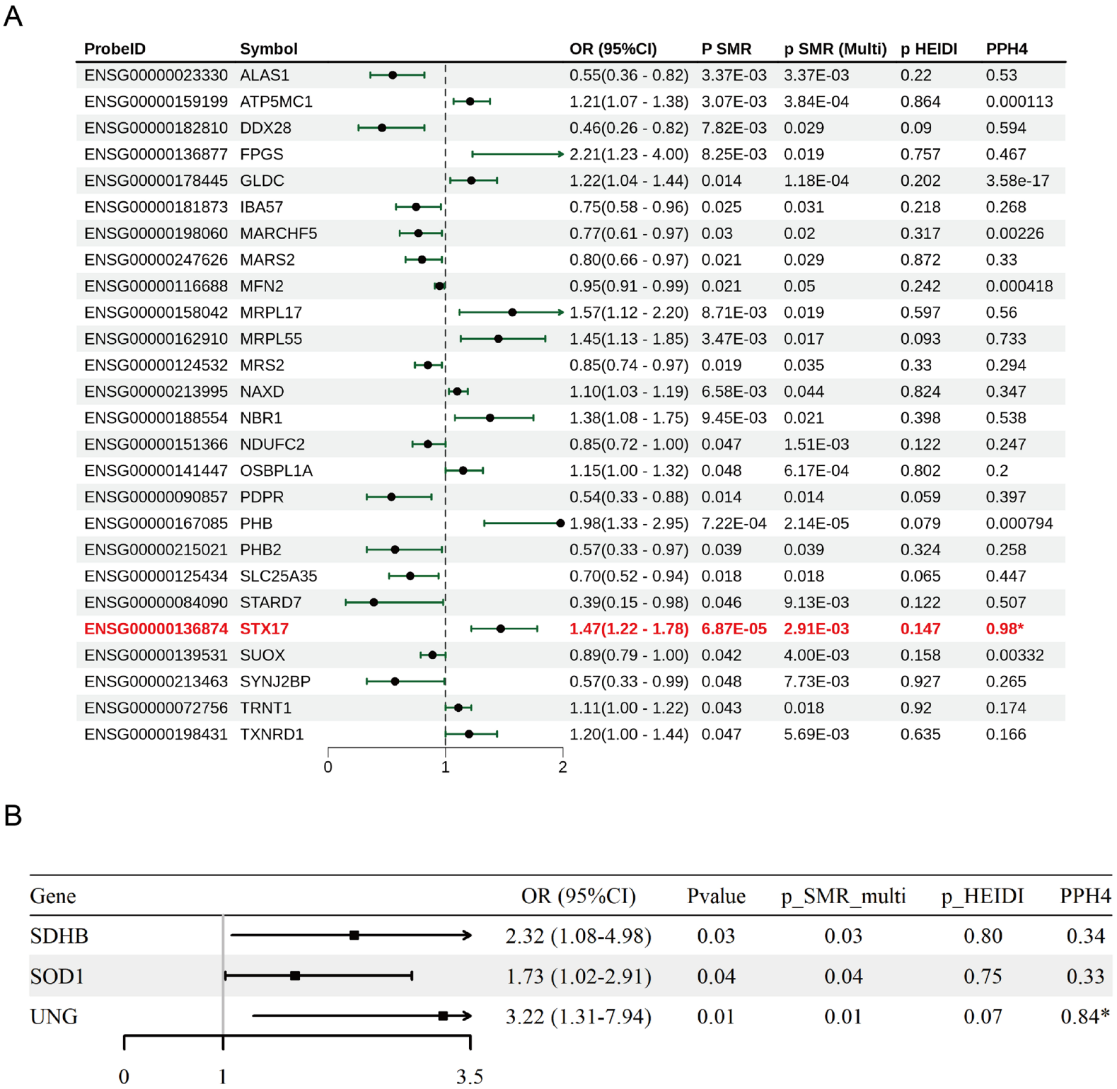
Forest plot. (A) SMR analysis results for eQTLs in the Finngen_R10_ASTHMA_CHILD_EXMORE cohort; (B) SMR analysis results for pQTLs in the Finngen_R10_ASTHMA_CHILD_EXMORE cohort.

SMR analysis linked 26 mitochondrial genes to asthma susceptibility (Figure 3A, Table S4). Twelve genes, including *NAXD*, *TRNT1*, *OSBPL1A*, *TXNRD1*, *ATP5MC1*, *GLDC*, *NBR1*, *MRPL55*, *STX17*, *MRPL17*, *PHB* and *FPGS*, showed positive correlations with asthma risk, while others demonstrated inverse associations. Seven genes showed strong colocalization (PP.H4 > 0.5; Figure S1B). *STX17* remained significant after FDR correction (FDR = 0.021) and exhibited high colocalization probability (PP.H4 = 0.98; Figure 3A). While exploratory replication in the GCST007800 dataset did not yield significant results (Table S5), *SUOX* and *SYNJ2BP* were replicated in the UKB‐D‐ASTHMA_CHILD cohort (Table S6).

### 3.3 Integration of Paediatric Asthma GWAS With Blood Mitochondrial‐Related pQTL Data

SMR analysis of protein abundance identified *SDHB*, *SOD1* and *UNG* as positively associated with asthma risk (Figure 3B, Table S7). *UNG* exhibited strong colocalization evidence ($PP.H4 = 0.84$). However, exploratory replication failed to identify these associations in either the GCST007800 or UKB‐D‐ASTHMA_CHILD datasets (Tables S8 and S9).

### 3.4 Multi‐Omics Evidence Integration

**FIGURE 4:**
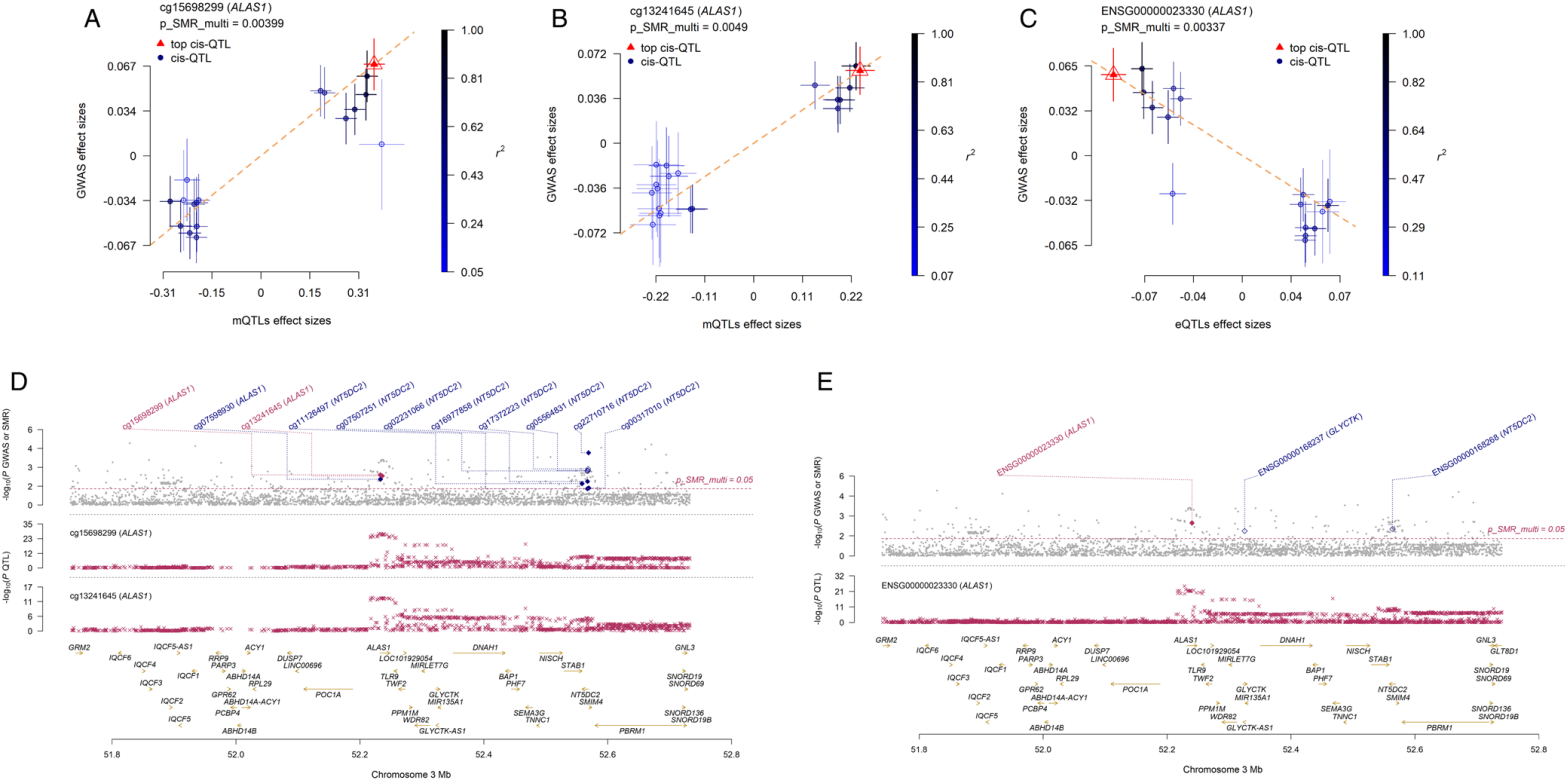
SMR locus plot and smr effect plot for *ALAS1*. (A) SMR effect plot for *ALAS1* at cg15698299 in mQTL; (B) SMR effect plot for *ALAS1* at cg13241645 in mQTL; (C) SMR effect plot of *ALAS1* in eQTL; (D) The SMR locus plot for *ALAS1* in mQTL; (E) The SMR locus plot for *ALAS1* in eQTL.

**FIGURE 5:**
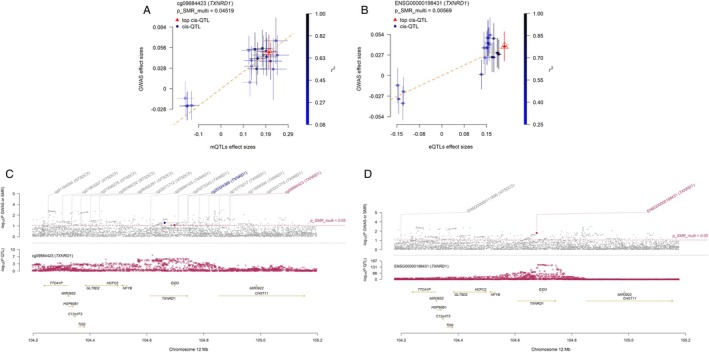
SMR locus plot and SMR effect plot for *TXNRD1*. (A) SMR effect plot for *TXNRD1* at cg09884423 in mQTL; (B) The SMR locus plot for *TXNRD1* in mQTL; (C) SMR effect plot of *TXNRD1* in eQTL; (D) The SMR locus plot for *TXNRD1* in eQTL.

**FIGURE 6:**
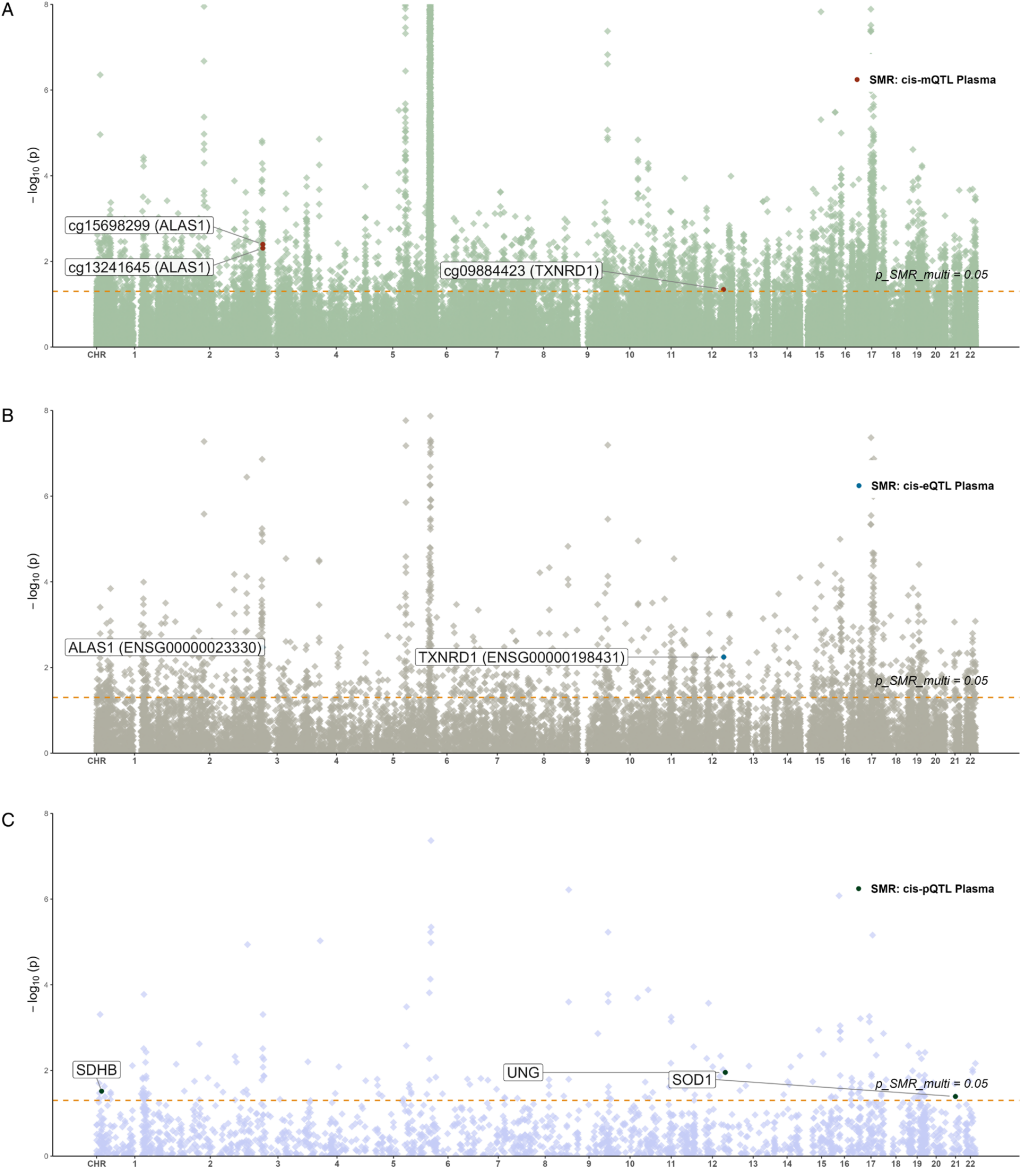
Manhattan plot of the distribution of key results on chromosomes. (A) cis‐mQTLs; (B) cis‐eQTLs; (C) cis‐pQTLs.

Integrated mQTL, eQTL and GWAS analysis revealed that *ALAS1*, *TXNRD1*, and *NBR1* associate with paediatric asthma across two molecular levels. Secondary SMR analysis (mQTL as exposure, eQTL as outcome) indicated that methylation at *ALAS1* (cg13241645, cg15698299) potentially downregulates gene expression, whereas *TXNRD1* methylation (cg09884423) correlates with increased expression (Table S10). Specifically, *ALAS1* methylation was associated with higher paediatric asthma risk and strong colocalization (PP.H4 > 0.8), while its expression correlated with lower risk and had moderate evidence (PP.H4 = 0.53; Figure S2; Figure 4). Similarly, *TXNRD1* methylation (cg09884423) was associated with higher risk, whereas its expression was associated with lower risk (Figure 5). No positive integrative results were observed for pQTLs; thus, eQTL and pQTL data were not combined in further SMR analyses. These findings collectively suggest that reduced methylation at cg09884423 may suppress *TXNRD1* expression and lower asthma risk, while increased methylation at cg13241645 and cg15698299 may downregulate *ALAS1*, potentially increasing disease risk. The genomic distribution of these significant associations across chromosomes is visualized in Figure 6.

### 3.5 Tissue‐Specific Validation and Replication in Independent Cohorts

To further confirm these associations, we performed an exploratory replication of gene expression levels in relevant tissues. Among the blood eQTL‐GWAS candidates, five genes (*FPGS, IBA57, NBR1, NDUFC2* and *STX17*) showed significant associations in tissue‐specific SMR analyses (Table S11).

### 3.6 Biological Functions, Interaction Network and Potential Drug Targets of Candidate Genes

To further understand the collective biological role of the 29 candidate genes identified from eQTL and pQTL analyses, we performed functional enrichment and network analyses.

The GO and KEGG enrichment results showed that the candidate genes were significantly enriched in mitochondria‐related functions. Key enriched KEGG pathways included ‘Oxidative phosphorylation’, ‘Mitophagy—animal’, and ‘Glycine, serine and threonine metabolism’. Significant GO terms were similarly related to core mitochondrial processes, such as ‘ATP metabolic process’ (BP), ‘mitochondrial inner membrane’ (CC) and ‘respiratory chain complex’ (CC) (Figure S3 and Tables S12 and S13).

A PPI network was constructed to map the interactions among the 29 candidate genes. Subsequent topological analysis identified seven Hub genes: *SDHB, MFN2, GLDC, PHB2, TXNRD1, ATP5MC1* and *PHB* (Figure S4 and Table S14). These genes occupy central positions in the network, suggesting they may play crucial regulatory roles.

To explore the therapeutic potential of these Hub genes, we performed a drug prediction analysis using the DSigDB database. The results identified several existing drugs and compounds that may target these key proteins. For example, genistein was significantly associated with multiple Hub genes (*GLDC, TXNRD1, MFN2, SDHB*), while aspirin was linked to *TXNRD1, PHB*, and *PHB2*. These findings provide preliminary leads for drug repurposing strategies (Full results in Table S15).

## 4 Discussion

In this work, we applied a multi‐omics MR framework to dissect the potential causal links between mitochondrial genes and paediatric asthma risk. Through comprehensive SMR analysis, we pinpointed 80 significant mQTLs (spanning 54 genes), 26 notable eQTLs, and three relevant pQTLs correlated with the disease. Through systematic replication in independent cohorts and integrative analysis across omics layers, *ALAS1* and *TXNRD1* emerged as functionally relevant candidate genes whose roles in asthma susceptibility warrant further experimental replication in independent cohorts. Importantly, further bioinformatics investigation suggested that these genes function within interconnected modules, highlighting their involvement in essential mitochondrial biological processes rather than acting individually.

The *ALAS1* gene, responsible for encoding the enzyme δ‐aminolevulinate synthase 1, is a key rate‐limiting enzyme in the heme synthesis pathway. Its protein product is located in the mitochondrial matrix and collaborates with other enzymes to promote heme production [28]. Studies have shown that the intracellular degradation of *ALAS1* is intrinsically linked to mitochondrial functionality and is controlled by ATP‐dependent proteases within the mitochondria [29]. While our genetic and epigenetic analyses suggest a potential role for *ALAS1* in paediatric asthma risk, it is important to note that these findings are based on statistical associations and in silico analyses; direct experimental evidence remains lacking. Our results indicate that lower *ALAS1* gene expression is linked to an increased risk of paediatric asthma, implying that insufficient *ALAS1* may result in deficient heme production, thereby disrupting mitochondrial function and potentially contributing to the development of asthma in children [30]. However, we emphasize that *ALAS1* should be considered a candidate gene pending further functional replication in independent cohorts.

The *TXNRD1* gene encodes thioredoxin reductase 1, an enzyme that is essential for maintaining mitochondrial redox homeostasis [31]. Notably, our PPI network analysis identified *TXNRD1* as a key Hub gene, underscoring its potential central regulatory role in the pathobiology of paediatric asthma. While current research has yet to establish a direct link between *TXNRD1* and asthma, given that oxidative stress is a significant factor in the pathogenesis of asthma [32], *TXNRD1*, as a key enzyme in redox regulation, may indirectly participate in the pathological process of asthma by affecting oxidative stress and inflammatory responses. Previous studies lend support to our hypothesis, suggesting that *TXNRD1* could participate in cigarette smoke‐induced oxidative stress and inflammation by modulating the activation of the Nrf2/HO‐1 signalling pathway [33]. Nevertheless, our methylation‐expression‐disease findings for TXNRD1, while compelling, remain hypothetical and should be interpreted as hypotheses for future functional studies rather than definitive mechanistic conclusions.

The functional enrichment analysis provides a broader biological context, reinforcing the central theme of this study. The significant enrichment of candidate genes in pathways like ‘Oxidative phosphorylation’, ‘ATP metabolic process’, and ‘Mitophagy—animal’ strongly supports the hypothesis that systemic mitochondrial dysfunction, beyond single gene effects, contributes to asthma risk. This concept is further solidified by the identification of a highly interconnected PPI network. The seven hub genes (*SDHB, MFN2, GLDC, PHB2, TXNRD1, ATP5MC1* and *PHB*) form the core of this network, suggesting that the genetic risk for paediatric asthma may be mediated through a coordinated disruption of mitochondrial quality control (*MFN2, PHB/PHB2*), energy production (*SDHB, ATP5MC1*) and redox balance (*TXNRD1*). The disruption of these central nodes could have cascading effects throughout the mitochondrial system, leading to the cellular dysfunction characteristic of asthma.

Additionally, tissue‐specific validation and replication in independent cohorts of this study identified genes such as *FPGS*, *IBA57*, *NBR1*, *NDUFC2* and *STX17* as being associated with asthma in lung tissue. Among them, *FPGS* is a key enzyme in folate metabolism [34], and folate is an essential cofactor for mitochondrial DNA synthesis and repair [35]. Abnormalities in the *FPGS* gene may affect mitochondrial function, thereby influencing airway inflammation and oxidative stress responses in asthma patients. The *IBA57* gene encodes a protein involved in the mitochondrial Fe/S cluster assembly process, which plays a key role in the activity of various mitochondrial enzymes [36]. Genetic mutations in *NDUFC2* may result in mitochondrial dysfunction, potentially influencing asthma susceptibility. NDUFC2 encodes a component of mitochondrial complex I, which plays a critical role in the mitochondrial respiratory chain [37]. Variations in the *NDUFC2* gene may disrupt mitochondrial energy metabolism and influence the inflammatory process of asthma. Both *NBR1* and *STX17* are involved in the process of mitochondrial autophagy. NBR1 is a selective autophagy receptor [38] and participates in mitochondrial autophagy in concert with p62 [39]. Abnormalities in the *NBR1* gene may affect mitochondria, which in turn affects the pathology of asthma. *STX17* is involved in mitochondrial autophagy, which helps to clear damaged mitochondria [40]. Abnormalities in the *STX17* gene could lead to impaired mitochondrial quality control, which in turn affects asthma.

Our drug prediction analysis is based entirely on in silico predictions using the DSigDB database and should not be interpreted as direct therapeutic recommendations. The identification of compounds such as genistein and aspirin, which are associated with several hub genes, provides only hypothesis‐generating leads for drug repurposing. It is important to note that aspirin can exacerbate respiratory symptoms in patients with aspirin‐exacerbated respiratory disease (AERD); therefore, any clinical application would require extreme caution and further experimental replication in independent cohorts. Our findings regarding aspirin should be interpreted as implicating possible involvement of salicylate‐related pathways in mitochondrial regulation, rather than direct endorsement of aspirin as a treatment for asthma. For instance, genistein—a soy‐derived isoflavone—was predicted to interact with multiple hub genes, including *GLDC, TXNRD1, MFN2* and *SDHB*. Genistein is known for its anti‐inflammatory and antioxidant properties, which align with the pathological mechanisms of asthma [41]. All drug predictions in this study should be regarded as preliminary and require future functional studies for replication in independent cohorts [42].

The multi‐omics MR method employed in this study overcomes the problems of confounding factors and reverse causality present in traditional epidemiological research, providing a more accurate assessment of causal effects. Through the integration of GWAS, mQTL, eQTL and pQTL datasets, this study assessed the influence of mitochondrial‐associated genes on paediatric asthma across multiple biological layers, shedding light on how mitochondrial dysfunction contributes to disease development [43]. Through colocalization analysis, the study was able to identify shared genetic variants that may influence paediatric asthma, further validating the genes with potential causal associations [22].

We performed exploratory replication of our main findings in two independent cohorts (GCST007800 and UKB‐d‐ASTHMA_CHILD). While some mQTL signals were successfully replicated, most eQTL and pQTL findings did not replicate in the GCST007800 dataset. In the UK Biobank validation cohort, the number of paediatric asthma cases (*n* = 1993) was relatively small, which resulted in reduced statistical power and may explain the lack of replication for some associations. Therefore, the absence of replication should be interpreted with caution and may reflect limited statistical power rather than false positive findings. Additionally, it is important to note that most available eQTL and pQTL datasets are derived from blood, and there is a lack of large‐scale, disease‐relevant tissue data (such as airway epithelium or lung immune cells), which further constrains the replication of our results in disease‐specific contexts.

Several limitations of this study should be acknowledged. First, our analysis was primarily based on European‐ancestry populations, consistent with the current availability of large‐scale GWAS datasets. While this approach ensures a relatively homogeneous genetic background and helps reduce population stratification bias, it also limits the generalizability of our findings to other ancestries and highlights the need for future multi‐ethnic studies. Second, we defined paediatric asthma using broad diagnostic criteria in order to maximize sample size and statistical power. However, this may obscure subtype‐specific mechanisms, such as differences between allergic and non‐allergic asthma, and future studies with more refined phenotyping are warranted. Third, standard nuclear GWAS arrays have limited coverage of mtDNA variants, which is a technical limitation inherent to current GWAS platforms. Dedicated sequencing studies focusing on mtDNA variation are needed to complement our findings. Fourth, although we implemented stringent HEIDI testing (*p* > 0.05) to filter out signals likely due to linkage rather than pleiotropy, the possibility of residual horizontal pleiotropy cannot be fully excluded, as is the case for all MR approaches. Despite these limitations, our study provides novel multi‐omics evidence for the involvement of mitochondrial‐related genes in paediatric asthma and lays the groundwork for future functional and translational investigations.

Future research could explore the functional mechanisms of these genes and develop targeted therapeutic strategies for mitochondrial function. For example, developing inhibitors or activators for the *ALAS1* and *TXNRD1* genes to treat paediatric asthma by controlling heme synthesis and redox capacity. Developing drugs targeting mitochondrial function, such as antioxidants and mitochondrial function enhancers, could improve mitochondrial function in paediatric asthma patients and alleviate asthma symptoms.

This study suggests the causal association between mitochondrial‐related genes and paediatric asthma, particularly the *ALAS1* and *TXNRD1* genes. Continued investigation into the involvement of mitochondrial‐related genes in paediatric asthma pathogenesis, along with the exploration of therapies aimed at modulating mitochondrial function, may offer novel strategies and insights for the precise prevention and management of paediatric asthma.

## Author Contributions

**Yanming Lu:** writing – original draft, writing – review and editing, visualization, methodology, validation, conceptualization. **Biyu Zhang:** conceptualization, writing – original draft, writing – review and editing, validation, methodology, formal analysis. **Yaqin Li:** writing – original draft, writing – review and editing, validation, methodology, software, formal analysis. **Bo Ding:** conceptualization, investigation, funding acquisition, visualization, formal analysis, data curation. **Xiaoyan Li:** data curation, formal analysis, methodology, writing – original draft, writing – review and editing.

## Funding

The authors have nothing to report.

## Ethics Statement

The authors have nothing to report.

## Consent

The authors have nothing to report.

## Conflicts of Interest

The authors declare no conflicts of interest.

## Supporting information


**Figure S1:** Forest Plots of mQTL‐ and eQTL–Paediatric Asthma Associations with Colocalization Evidence. (A) Forest plot of methylation sites (mQTLs) significantly associated with paediatric asthma risk (SMR *p* < 0.05, multi‐SNP SMR *p* < 0.05, HEIDI *p* > 0.05) and strong colocalization evidence (PP.H4 > 0.5). Each row represents a methylation site (CpG) annotated to its gene, with odds ratios (OR) and 95% confidence intervals (CI) displayed. Only sites passing the stringent colocalization threshold are shown. (B) Forest plot of mitochondrial‐related gene expression (eQTLs) significantly associated with paediatric asthma risk (SMR *p* < 0.05, multi‐SNP SMR *p* < 0.05, HEIDI *p* > 0.05). Genes with strong colocalization evidence (PP.H4 > 0.5) are highlighted in red, while those with moderate or lower evidence (PP.H4 < 0.8) are shown in black. Odds ratios and 95% confidence intervals are indicated for each gene.

**Figure S2:** Colocalization Results. (A) mQTLs associated with Finngen_R10_ASTHMA_CHILD_EXMORE Cohort for *ALAS1* at cg15698299; (B) mQTLs associated with Finngen_R10_ASTHMA_CHILD_EXMORE Cohort for *ALAS1* at cg13241645. C. eQTLs associated with Finngen_R10_ASTHMA_CHILD_EXMORE Cohort for *ALAS1*.

**Figure S3:** GO and KEGG annotation plot.

**Figure S4:** PPI network plot with Hub genes highlighted.

**Tables S1:** Finngen_R10_ASTHMA_CHILD_EXMORE Cohort mQTLs‐GWAS SMR Analysis Results.
**Tables S2:** mQTLs‐GWAS Validation Results: mQTLs SMR Analysis by GCST007800 Cohort
**Tables S3:** mQTLs‐GWAS Validation Results: mQTLs SMR Analysis by UKB‐d‐ASTHMA_CHILD Cohort.
**Tables S4:** Finngen_R10_ASTHMA_CHILD_EXMORE Cohort eQTLs‐GWAS SMR Analysis Results.
**Tables S5:** eQTLs‐GWAS Validation Results: eQTLs SMR Analysis by GCST007800 Cohor.
**Tables S6:** eQTLs‐GWAS Validation Results: eQTLs SMR Analysis by UKB‐d‐ASTHMA_CHILD Cohort.
**Tables S7:** Finngen_R10_ASTHMA_CHILD_EXMORE Cohort pQTLs‐GWAS SMR Analysis Results.
**Tables S8:** pQTLs‐GWAS Validation Results: pQTLs SMR Analysis by GCST007800 Cohort.
**Tables S9:** pQTLs‐GWAS Validation Results: pQTLs SMR Analysis by UKB‐d‐ASTHMA_CHILD Cohort.
**Tables S10:** mQTLs‐ eQTLs SMR Analysis Results.
**Tables S11:** SMR Analysis of eQTLs in GTEx Lung Data with Finngen_R10_ASTHMA_CHILD_EXMORE cohort.
**Table S12:** Detailed Gene Ontology enrichment results.
**Table S13:** Detailed KEGG pathway enrichment results.
**Table S14:** Full CentiScaPe analysis results for all 29 genes.
**Table S15:** Full DSigDB drug prediction results.

## Data Availability

All data generated or analysed during this study are included in this published article.

## References

[1] L. A. Conrad , M. D. Cabana , and D. Rastogi , “Defining Pediatric Asthma: Phenotypes to Endotypes and Beyond,” Pediatric Research 90, no. 1 (2021): 45–51. 33173175 10.1038/s41390-020-01231-6 PMC8107196

[2] P. M. Pitrez , S. Nanthapisal , A. Castro , C. Teli , and P. G. Abhijith , “Managing Moderate‐To‐Severe Paediatric Asthma: A Scoping Review of the Efficacy and Safety of Fluticasone Propionate/Salmeterol,” BMJ Open Respiratory Research 10, no. 1 (2023): 1–8, 10.1136/bmjresp-2023-001706. PMC10450074 37620110

[3] J. López‐Tiro , A. Contreras‐Contreras , M. E. Rodríguez‐Arellano , and P. Costa‐Urrutia , “Economic Burden of Severe Asthma Treatment: A Real‐Life Study,” World Allergy Organization Journal 15, no. 7 (2022): 100662. 35833203 10.1016/j.waojou.2022.100662 PMC9260620

[4] C. Nunes , A. M. Pereira , and M. Morais‐Almeida , “Asthma Costs and Social Impact,” Asthma Research and Practice 3 (2017): 1. 28078100 10.1186/s40733-016-0029-3 PMC5219738

[5] L. Qian , E. Mehrabi Nasab , S. M. Athari , and S. S. Athari , “Mitochondria Signaling Pathways in Allergic Asthma,” Journal of Investigative Medicine 70, no. 4 (2022): 863–882. 35168999 10.1136/jim-2021-002098 PMC9016245

[6] M. P. Rossmann , S. M. Dubois , S. Agarwal , and L. I. Zon , “Mitochondrial Function in Development and Disease,” Disease Models & Mechanisms 14, no. 6 (2021): 1–36. 34114603 10.1242/dmm.048912 PMC8214736

[7] V. S. Ten and V. Ratner , “Mitochondrial Bioenergetics and Pulmonary Dysfunction: Current Progress and Future Directions,” Paediatric Respiratory Reviews 34 (2020): 37–45. 31060947 10.1016/j.prrv.2019.04.001 PMC6790157

[8] M. L. Tsai , Y. G. Tsai , Y. C. Lin , et al., “IL‐25 Induced ROS‐Mediated M2 Macrophage Polarization via AMPK‐Associated Mitophagy,” International Journal of Molecular Sciences 23, no. 1 (2021): 1–18. 35008429 10.3390/ijms23010003 PMC8744791

[9] K. Sachdeva , D. C. Do , Y. Zhang , X. Hu , J. Chen , and P. Gao , “Environmental Exposures and Asthma Development: Autophagy, Mitophagy, and Cellular Senescence,” Frontiers in Immunology 10 (2019): 2787. 31849968 10.3389/fimmu.2019.02787 PMC6896909

[10] Y. C. Lin , Y. C. Lin , M. L. Tsai , W. T. Liao , and C. H. Hung , “TSLP Regulates Mitochondrial ROS‐Induced Mitophagy via Histone Modification in Human Monocytes,” Cell & Bioscience 12, no. 1 (2022): 32. 35292112 10.1186/s13578-022-00767-w PMC8925056

[11] M. P. Cocco , E. White , S. Xiao , et al., “Asthma and Its Relationship to Mitochondrial Copy Number: Results From the Asthma Translational Genomics Collaborative (ATGC) of the Trans‐Omics for Precision Medicine (TOPMed) Program,” PLoS One 15, no. 11 (2020): e0242364. 33237978 10.1371/journal.pone.0242364 PMC7688161

[12] A. Flaquer , A. Heinzmann , S. Rospleszcz , et al., “Association Study of Mitochondrial Genetic Polymorphisms in Asthmatic Children,” Mitochondrion 14, no. 1 (2014): 49–53. 24270090 10.1016/j.mito.2013.11.002

[13] C. Xulong , Z. Li , and Y. Tongjin , “The Effects of NLRP3 and MAVS Gene Polymorphisms on the Risk of Asthma: A Case‐Control Study,” Medicine (Baltimore) 101, no. 51 (2022): e32385. 36595748 10.1097/MD.0000000000032385 PMC9794206

[14] Z. Wang , M. Li , Q. Zhou , and Y. Shang , “Protective Effects of a Neurokinin 1 Receptor Antagonist on Airway Epithelial Mitochondria Dysfunction in Asthmatic Mice via Nrf2/HO‐1 Activation,” International Immunopharmacology 77 (2019): 105952. 31677499 10.1016/j.intimp.2019.105952

[15] U. Võsa , A. Claringbould , H. J. Westra , et al., “Large‐Scale Cis‐ and Trans‐eQTL Analyses Identify Thousands of Genetic Loci and Polygenic Scores That Regulate Blood Gene Expression,” Nature Genetics 53, no. 9 (2021): 1300–1310. 34475573 10.1038/s41588-021-00913-z PMC8432599

[16] Y. Wu , J. Zeng , F. Zhang , et al., “Integrative Analysis of Omics Summary Data Reveals Putative Mechanisms Underlying Complex Traits,” Nature Communications 9, no. 1 (2018): 918. 10.1038/s41467-018-03371-0 PMC5834629 29500431

[17] S. Xu , J. Liang , T. Shen , D. Zhang , and Z. Lu , “Causal Links Between Immune Cells and Asthma: Insights From a Mendelian Randomization Analysis,” Journal of Asthma 62, no. 2 (2025): 346–353. 10.1080/02770903.2024.2403740 39269201

[18] J. Wang , J. Hu , D. Qin , D. Han , and J. Hu , “A Multi‐Omics Mendelian Randomization Identifies Putatively Causal Genes and DNA Methylation Sites for Asthma,” World Allergy Organization Journal 17, no. 12 (2024): 101008. 39720783 10.1016/j.waojou.2024.101008 PMC11667005

[19] P. Sekula , M. F. Del Greco , C. Pattaro , and A. Köttgen , “Mendelian Randomization as an Approach to Assess Causality Using Observational Data,” Journal of the American Society of Nephrology: JASN 27, no. 11 (2016): 3253–3265. 27486138 10.1681/ASN.2016010098 PMC5084898

[20] B. B. Sun , J. Chiou , M. Traylor , et al., “Plasma Proteomic Associations With Genetics and Health in the UK Biobank,” Nature 622, no. 7982 (2023): 329–338. 37794186 10.1038/s41586-023-06592-6 PMC10567551

[21] T. Qi , Y. Wu , J. Zeng , et al., “Identifying Gene Targets for Brain‐Related Traits Using Transcriptomic and Methylomic Data From Blood,” Nature Communications 9, no. 1 (2018): 2282. 10.1038/s41467-018-04558-1 PMC5995828 29891976

[22] A. Hukku , M. Pividori , F. Luca , R. Pique‐Regi , H. K. Im , and X. J. Wen , “Probabilistic Colocalization of Genetic Variants From Complex and Molecular Traits: Promise and Limitations,” American Journal of Human Genetics 108, no. 1 (2021): 25–35. 33308443 10.1016/j.ajhg.2020.11.012 PMC7820626

[23] E. Pairo‐Castineira , K. Rawlik , A. D. Bretherick , et al., “GWAS and Meta‐Analysis Identifies 49 Genetic Variants Underlying Critical COVID‐19,” Nature 617, no. 7962 (2023): 764–768. 37198478 10.1038/s41586-023-06034-3 PMC10208981

[24] Z. Zhu , F. Zhang , H. Hu , et al., “Integration of Summary Data From GWAS and eQTL Studies Predicts Complex Trait Gene Targets,” Nature Genetics 48, no. 5 (2016): 481–487. 27019110 10.1038/ng.3538

[25] W. Hu and Y. Xu , “Transcriptomics in Idiopathic Pulmonary Fibrosis Unveiled: A New Perspective From Differentially Expressed Genes to Therapeutic Targets,” Frontiers in Immunology 15 (2024): 1375171. 38566986 10.3389/fimmu.2024.1375171 PMC10985171

[26] S. Shi , X. Tian , Y. Gong , et al., “Pivotal Role of JNK Protein in the Therapeutic Efficacy of Parthenolide Against Breast Cancer: Novel and Comprehensive Evidences From Network Pharmacology, Single‐Cell RNA Sequencing and Metabolomics,” International Journal of Biological Macromolecules 279, no. Pt 3 (2024): 135209. 39244135 10.1016/j.ijbiomac.2024.135209

[27] M. Yoo , J. Shin , J. Kim , et al., “DSigDB: Drug Signatures Database for Gene Set Analysis,” Bioinformatics 31, no. 18 (2015): 3069–3071. 25990557 10.1093/bioinformatics/btv313 PMC4668778

[28] L. S. Dunaway , S. A. Loeb , S. Petrillo , E. Tolosano , and B. E. Isakson , “Heme Metabolism in Nonerythroid Cells,” Journal of Biological Chemistry 300, no. 4 (2024): 107132. 38432636 10.1016/j.jbc.2024.107132 PMC10988061

[29] Y. Kubota , K. Nomura , Y. Katoh , R. Yamashita , K. Kaneko , and K. Furuyama , “Novel Mechanisms for Heme‐Dependent Degradation of ALAS1 Protein as a Component of Negative Feedback Regulation of Heme Biosynthesis*,” Journal of Biological Chemistry 291, no. 39 (2016): 20516–20529. 27496948 10.1074/jbc.M116.719161 PMC5034046

[30] S. A. Swenson , C. M. Moore , J. R. Marcero , A. E. Medlock , A. R. Reddi , and O. Khalimonchuk , “From Synthesis to Utilization: The Ins and Outs of Mitochondrial Heme,” Cells 9, no. 3 (2020): 579. 32121449 10.3390/cells9030579 PMC7140478

[31] S. I. Oka , A. Chin , J. Y. Park , et al., “Thioredoxin‐1 Maintains Mitochondrial Function via Mechanistic Target of Rapamycin Signalling in the Heart,” Cardiovascular Research 116, no. 10 (2020): 1742–1755. 31584633 10.1093/cvr/cvz251 PMC7825501

[32] U. M. Sahiner , E. Birben , S. Erzurum , C. Sackesen , and O. Kalayci , “Oxidative Stress in Asthma,” World Allergy Organization Journal 4, no. 10 (2011): 151–158. 23268432 10.1097/WOX.0b013e318232389e PMC3488912

[33] Q. Huang , M. Peng , Y. Gu , et al., “Metabolism‐Related Gene TXNRD1 Regulates Inflammation and Oxidative Stress Induced by Cigarette Smoke Through the Nrf2/HO‐1 Pathway in the Small Airway Epithelium,” Oxidative Medicine and Cellular Longevity 2022 (2022): 7067623. 36578523 10.1155/2022/7067623 PMC9792251

[34] S. E. Kim , “Enzymes Involved in Folate Metabolism and Its Implication for Cancer Treatment,” Nutrition Research and Practice 14, no. 2 (2020): 95–101. 32256983 10.4162/nrp.2020.14.2.95 PMC7075736

[35] M. M. Zarou , A. Vazquez , and G. Vignir Helgason , “Folate Metabolism: A Re‐Emerging Therapeutic Target in Haematological Cancers,” Leukemia 35, no. 6 (2021): 1539–1551. 33707653 10.1038/s41375-021-01189-2 PMC8179844

[36] F. Zhan , X. Liu , R. Ni , et al., “Novel IBA57 Mutations in Two Chinese Patients and Literature Review of Multiple Mitochondrial Dysfunction Syndrome,” Metabolic Brain Disease 37, no. 2 (2022): 311–317. 34709542 10.1007/s11011-021-00856-8

[37] A. B. Bandara , J. C. Drake , C. C. James , J. W. Smyth , and D. A. Brown , “Complex I Protein NDUFS2 Is Vital for Growth, ROS Generation, Membrane Integrity, Apoptosis, and Mitochondrial Energetics,” Mitochondrion 58 (2021): 160–168. 33744462 10.1016/j.mito.2021.03.003 PMC8113094

[38] N. L. Rasmussen , A. Kournoutis , T. Lamark , and T. Johansen , “NBR1: The Archetypal Selective Autophagy Receptor,” Journal of Cell Biology 221, no. 11 (2022): 1–15. 10.1083/jcb.202208092 PMC9582228 36255390

[39] V. Kirkin , T. Lamark , T. Johansen , and I. Dikic , “NBR1 Cooperates With p62 in Selective Autophagy of Ubiquitinated Targets,” Autophagy 5, no. 5 (2009): 732–733. 19398892 10.4161/auto.5.5.8566

[40] H. Xian , Q. Yang , L. Xiao , H. M. Shen , and Y. C. Liou , “STX17 Dynamically Regulated by Fis1 Induces Mitophagy via Hierarchical Macroautophagic Mechanism,” Nature Communications 10, no. 1 (2019): 2059. 10.1038/s41467-019-10096-1 PMC6499814 31053718

[41] Y. X. Goh , J. Jalil , K. W. Lam , K. Husain , and C. M. Premakumar , “Genistein: A Review on Its Anti‐Inflammatory Properties,” Frontiers in Pharmacology 13 (2022): 820969. 35140617 10.3389/fphar.2022.820969 PMC8818956

[42] J. Mullur and K. M. Buchheit , “Aspirin‐Exacerbated Respiratory Disease: Updates in the Era of Biologics,” Annals of Allergy, Asthma & Immunology 131, no. 3 (2023): 317–324. 10.1016/j.anai.2023.05.016 PMC10524829 37225000

[43] C. Jin , B. Lee , L. Shen , and Q. Long , “Integrating Multi‐Omics Summary Data Using a Mendelian Randomization Framework,” Briefings in Bioinformatics 23, no. 6 (2022): 1–13. 10.1093/bib/bbac376 PMC9677504 36094096

