# Supplementary figures and images for: Mitochondria‐Related Pathogenic Genes in Paediatric Asthma: A Multi‐Omics Mendelian Randomization Study
Source: J Cell Mol Med. 2026 Mar 27;30(7):e71102. doi: 10.1111/jcmm.71102 (PMC13140575; doi:10.1111/jcmm.71102)

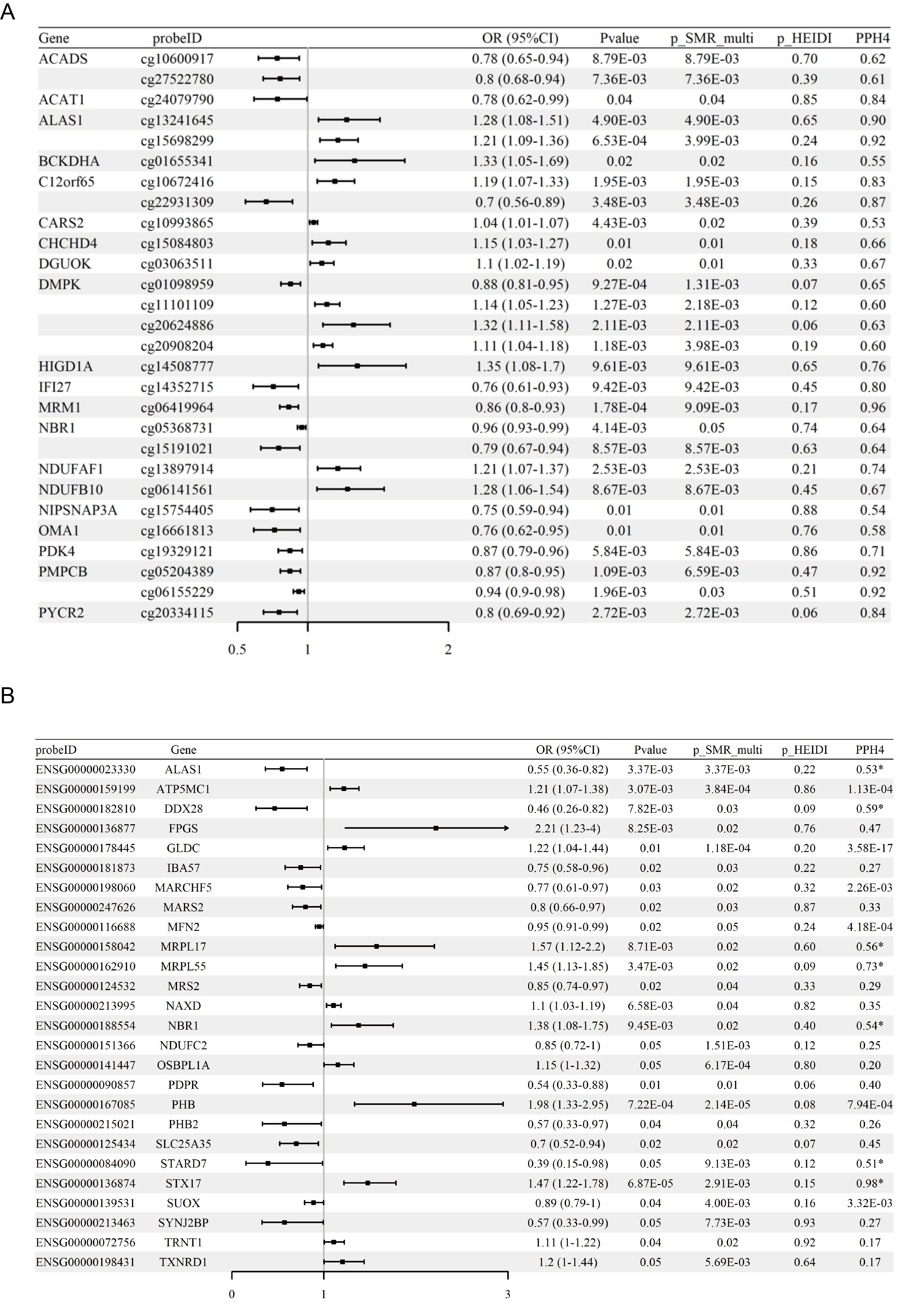

Supplement: Supplementary file 1 — Figure S1: Forest Plots of mQTL‐ and eQTL–Paediatric Asthma Associations with Colocalization Evidence. (A) Forest plot of methylation sites (mQTLs) significantly associated with paediatric asthma risk (SMR p < 0.05, multi‐SNP SMR p < 0.05, HEIDI p > 0.05) and strong colocalization evidence (PP.H4 > 0.5). Each row represents a methylation site (CpG) annotated to its gene, with odds ratios (OR) and 95% confidence intervals (CI) displayed. Only sites passing the stringent colocalization threshold are shown. (B) Forest plot of mitochondrial‐related gene expression (eQTLs) significantly associated with paediatric asthma risk (SMR p < 0.05, multi‐SNP SMR p < 0.05, HEIDI p > 0.05). Genes with strong colocalization evidence (PP.H4 > 0.5) are highlighted in red, while those with moderate or lower evidence (PP.H4 < 0.8) are shown in black. Odds ratios and 95% confidence intervals are indicated for each gene. [file JCMM-30-e71102-s003.jpg]

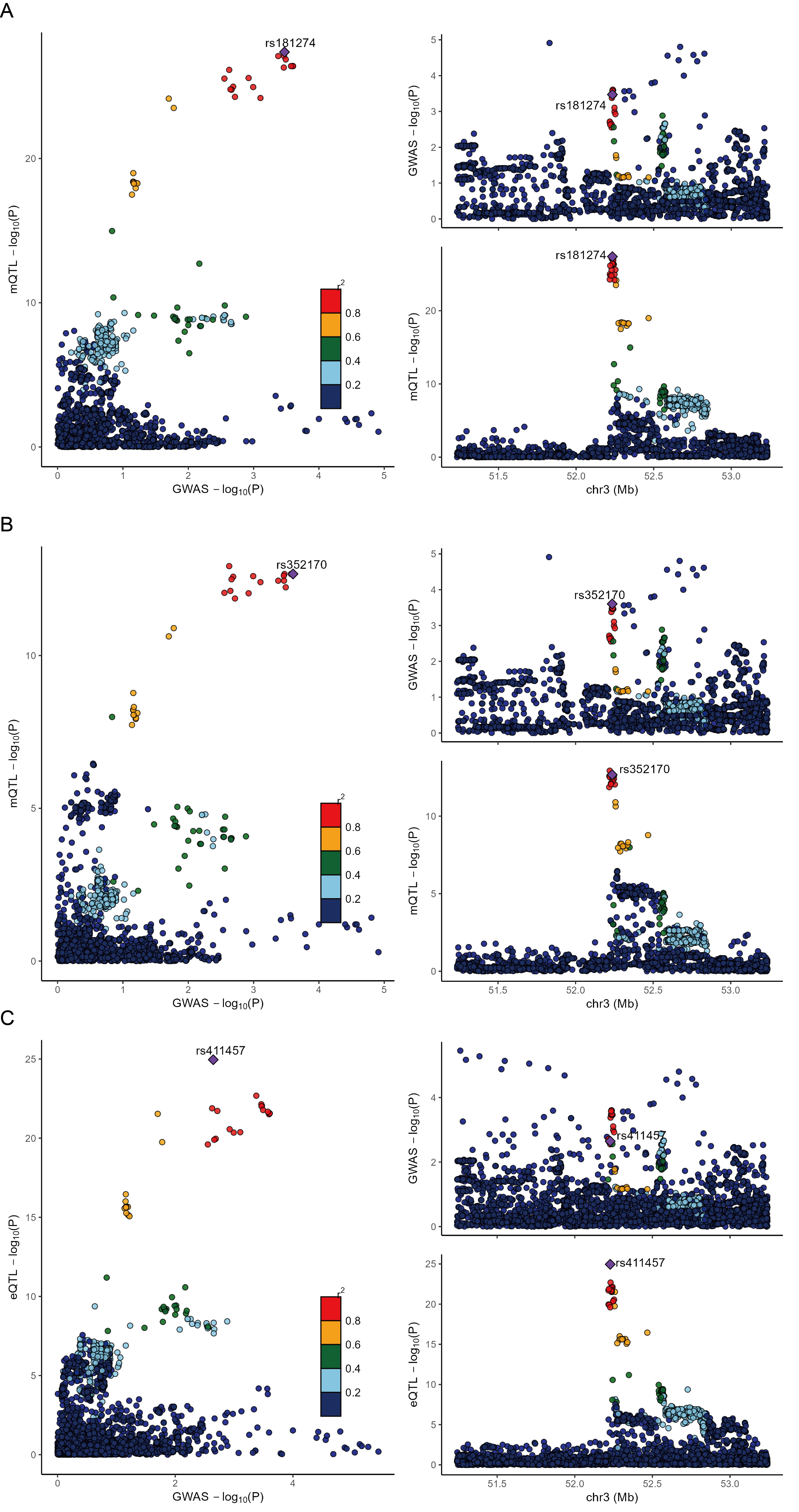

Supplement: Supplementary file 2 — Figure S2: Colocalization Results. (A) mQTLs associated with Finngen_R10_ASTHMA_CHILD_EXMORE Cohort for ALAS1 at cg15698299; (B) mQTLs associated with Finngen_R10_ASTHMA_CHILD_EXMORE Cohort for ALAS1 at cg13241645. C. eQTLs associated with Finngen_R10_ASTHMA_CHILD_EXMORE Cohort for ALAS1. [file JCMM-30-e71102-s002.jpg]

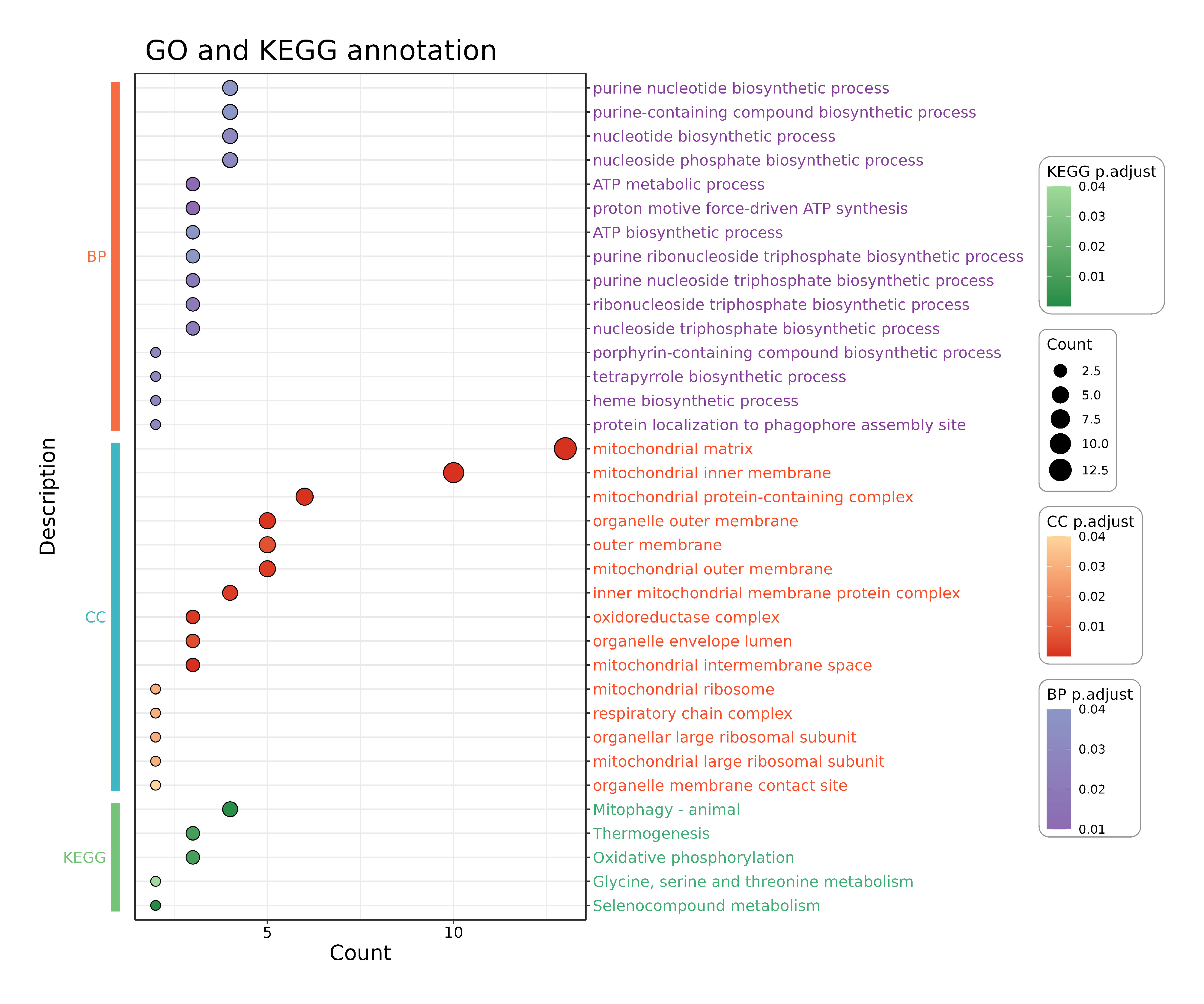

Supplement: Supplementary file 3 — Figure S3: GO and KEGG annotation plot. [file JCMM-30-e71102-s005.jpg]

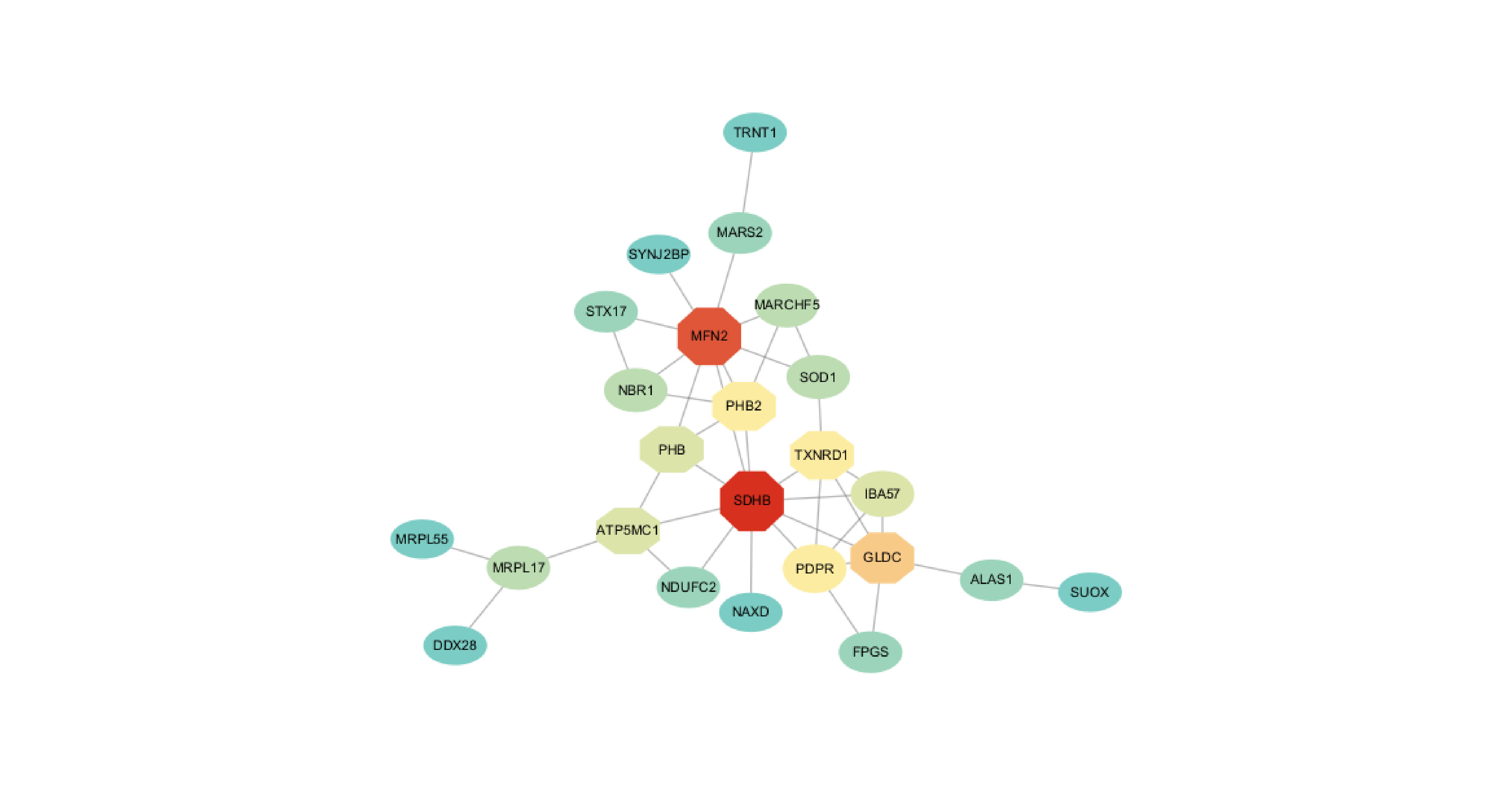

Supplement: Supplementary file 4 — Figure S4: PPI network plot with Hub genes highlighted. [file JCMM-30-e71102-s001.jpg]
